# Saunders’s Medical Hand-Atlases

**Published:** 1902-12

**Authors:** 


SAUNDERS'S MEDICAL HAND-ATLASES. Atlas and Epitomé of Abdominal Hernias. By Privat docent Dr. GEORG SULTAN, of Göttingen. Edited, with additions, by William B. Coley, M. D., Clinical Lecturer on Surgery. Columbia University (College of Physicians and Surgeons). With 119 illustrations, 36 of them in colors, and 277 pages of text. Philadelphia and London: W. B. Saunders & Co. 1902. [Cloth, $3.00 net.]

In his preface Dr. Sultan says that if this book should succeed in awakening an interest in abdominal hernias among physicians and students and incite them to a careful study of the subject he will consider that his labors have been rewarded. In that case he should rest satisfied, for one could not take up the book for even a casual running over without being deeply interested and studying it seriously. Its text is terse and interesting, and the illustrations are marvelously correct and clear to the understanding. The book edited by Dr. William B. Coley is sufficient evidence that every point regarding hernia in all its aspects is made understandable.

